# Voxel-Wise Meta-Analysis of Gray Matter Changes in Amyotrophic Lateral Sclerosis

**DOI:** 10.3389/fnagi.2016.00064

**Published:** 2016-03-30

**Authors:** Dongchao Shen, Liying Cui, Jia Fang, Bo Cui, Dawei Li, Hongfei Tai

**Affiliations:** ^1^Department of Neurology, Peking Union Medical College Hospital, Peking Union Medical College and Chinese Academy of Medical SciencesBeijing, China; ^2^Neuroscience Center, Chinese Academy of Medical SciencesBeijing, China

**Keywords:** amyotrophic lateral sclerosis, gray matter, voxel-based morphometry, meta-analysis, signed differential mapping

## Abstract

**Background:** Increasing neuroimaging studies have revealed gray matter (GM) anomalies of several brain regions by voxel-based morphometry (VBM) studies in patients with amyotrophic lateral sclerosis (ALS). A voxel-wise meta-analysis was conducted to integrate the reported studies to determine the consistent GM alterations in ALS based on VBM methods.

**Methods:** Ovid Medline, Pubmed, Emabase, and BrainMap database were searched for relevant studies.Data were extracted by two independent researchers. Voxel-wise meta-analysis was performed using the effect-size signed differential mapping (ES-SDM) software.

**Results:** Twenty-nine VBM studies comprising 638 subjects with ALS and 622 healthy controls (HCs) met inclusion criteria.The global GM volumes of ALS patients were significantly decreased compared with those of HCs. GM reductions in patients were mainly located in the right precentral gyrus, the left Rolandic operculum, the left lenticular nucleus and the right anterior cingulate/paracingulate gyri. The right precentral gyrus and the left inferior frontal gyrus might be potential anatomical biomarkers to evaluate the severity of the disease, and longer disease duration was associated with more GM atrophy in the left frontal aslant tract and the right precentral gyrus in ALS patients.

**Conclusion:** The results support that ALS is a complex degenerative disease involving multisystems besides the motor system.The mechanism of asymmetric atrophy of the motor cortex and the implication of Rolandic operculum involvement in ALS need to be further elucidated in future studies.

## Introduction

Amyotrophic lateral sclerosis (ALS) is a progressive neurodegenerative disease characterized by involvement of both upper motor neuron (UMN) and lower motor neuron (LMN), with a median survival time of 2–4 years from onset of symptoms in population-based studies (Beghi et al., 2006). The cause of this disease remains largely unknown, but there is an increasing awareness that the neurodegeneration of ALS is not only restricted to the motor system but also involves sensory, language, behavior, and other cognitive fields (Strong et al., 2009). As a fully-automated whole-brain technique, the voxel-based morphometry (VBM) method overcomes the limitations of region of interest (ROI) approach that requires a priori decision concerning which structures needed to be evaluated, therefore provides a powerful and unbiased tool to study the gray matter (GM) changes in ALS. Unfortunately, applications of this novel method in the study of ALS are often limited by relatively small sample sizes, resulting in insufficient statistical power and increased risk of false-positive results. Whereas an independent voxel-wise meta-analysis could further integrate VBM findings or other functional neuroimaging studies, and offer insights that are not apparent from individual studies and obtain consistent results across different studies.

A recently developed meta-analytic tool, namely signed differential mapping (SDM), has been increasingly used to summarize functional neuroimaging results (Radua and Mataix-Cols, 2009). The latest version called effect-size SDM (ES-SDM) combines various positive features of earlier meta-analytic methods, such as activation likelihood estimation and multilevel kernel density analysis, and has been effectively applied in the investigation of multiple disorders, such as obsessive-compulsive disorder (Radua and Mataix-Cols, 2009), major depressive disorder (Zhao et al., 2014), Alzheimer's disease (Wang et al., 2015), Parkinson's disease (Pan et al., 2012), and also in ALS (Chen and Ma, 2010). However, the previous meta-analysis of VBM studies in ALS which was performed in 2010 only included 5 studies (Chen and Ma, 2010). Furthermore, after comprehensive retrieve and careful data check, we found that no study with negative results was included (i.e., when no significant differences between individuals with ALS and controls were found) and that the authors might adopt the data of ALS patients with frontotemporal dementia (FTD) in one study (Chang et al., 2005). In both cases, the changes of GM in ALS patients would be overestimated. Therefore, the present work aims to update the voxel-wise meta-analysis of VBM studies in ALS using the latest ES-SDM software. In addition, this meta-analysis is also intended to explore the effect of clinical quantitative variables on the GM alternations in ALS through meta-regression.

## Methods

### Inclusion of studies

Ovid Medline, Pubmed and Embase databases were searched for studies published up to May 2015 that reported VBM data in patients with ALS. Search terms included “motor neuron disease,” “MND,” “amyotrophic lateral sclerosis,” “ALS,” and these terms were combined using the AND operator with “voxel-based,” “voxelwise,” “voxel-wise,” “morphometry,” “voxel-based morphometry,” “voxel based morphometry,” “VBM” and “gray matter.” Both text word and MeSH subject headings were used. Language was confined to English, and reviews were excluded in the advanced search. The BrainMap database (http://brainmap.org/sleuth/account.html), which is an online database of functional and structural neuroimaging results in the form of stereotactic (x, y, z) coordinates, was also searched for experiments of VBM in ALS. The search strategy was supplemented by inspecting the reference lists of included articles. The studies were considered for inclusion if they (1) reported VBM (GM density or volume) comparison between patients with ALS and healthy controls (HCs); (2) reported whole brain results of GM changes of ALS patients in Talairach or Montreal Neurological Institute (MNI) space. Studies were excluded if they were in line with the following criteria: (1) there was no HC group; (2) used a ROI approach; (3) the comparisons contained a subgroup of ALS-FTD; (4) did not list coordinates for the contrasts. When the same study population was reported in more than one article, the data were included only once. The authors of relevant studies were also contacted by email when necessary. Meta-analysis Of Observational Studies in Epidemiology (MOOSE) guidelines were followed in this analysis (Stroup et al., 2000).

### Data extraction

For each included study, the following data were extracted: numbers of participants, mean age, site of symptom onset, mean disease duration, mean ALS functional rating scale (ALSFRS) scores or revised ALSFRS (ALSFRS-R) scores and technical parameters of image scanning and analysis. The coordinates in each study were independently extracted by two researchers according to the ES-SDM method.

### Voxel-wise meta-analysis of included studies

Voxel-wise meta-analysis was performed using the ES-SDM software (version 4.31, http://www.sdmproject.com/). The methods have been described in detail elsewhere (Radua et al., 2012, 2014). The full width at half-maximum (FWHM) was set at 20 mm, which had excellent control for false positives according to previous studies; and the statistical threshold was set to be a *p* < 0.005 without correction for false discovery rate (FDR), which was found to be able to optimize the balance between sensitivity and specificity (Radua et al., 2012). Globals analysis, Mean analysis, and Jackknife sensitivity analysis were carried out. In order to control for possible methodological differences observed between included studies, the analysis was repeated several times including only those studies which are methodologically homogenous. The last analysis was a meta-regression of voxel values across the studies by disease severity (proportional scores, ALSFRS/40 or ALSFRS-R/48) and disease duration of the patients' samples.

## Results

### Included studies and sample characteristics

**Figure 1:**
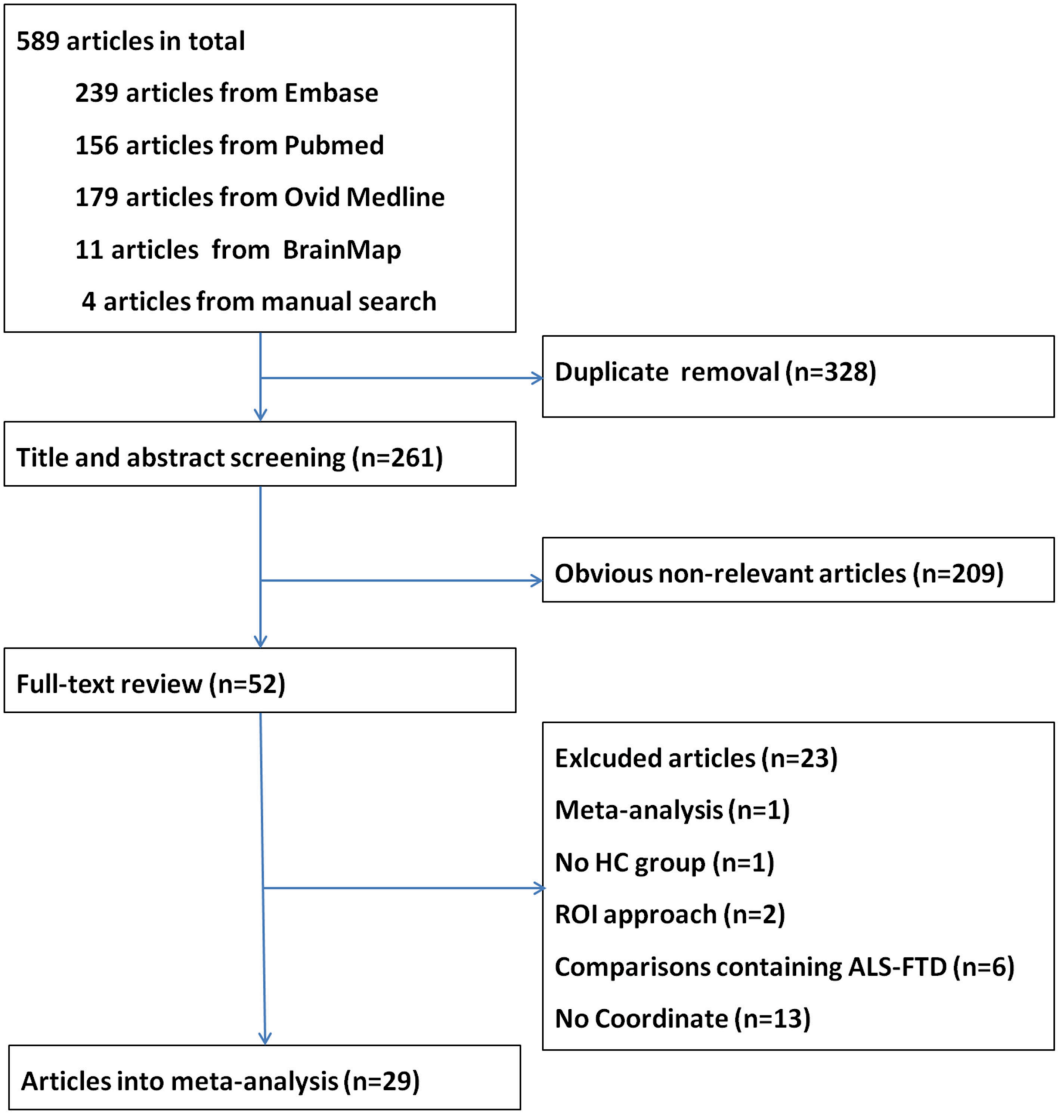
**The flow chart of the literature search in the meta-analysis**.

A total of 589 articles were identified. After removal of duplicate entries, 261 articles remained and then were screened by title and abstract. As a result, 209 were excluded because they focused on disorders other than MND, were animal experiments or case reports, or did not involve VBM. After full-text review, another 23 papers were excluded for different reasons. A flow chart of publication selection is presented in Figure 1. Finally, 29 studies met the selection criteria and had accessible information concerning GM changes between ALS and HC (Ellis et al., 2001; Abrahams et al., 2005; Chang et al., 2005; Grosskreutz et al., 2006; Agosta et al., 2007, 2009; Mezzapesa et al., 2007; Thivard et al., 2007; Grossman et al., 2008; Minnerop et al., 2009; Roccatagliata et al., 2009; Canu et al., 2011; Senda et al., 2011; Cosottini et al., 2012, 2013; Luo et al., 2012; Tedeschi et al., 2012; Kwan et al., 2013; Bede et al., 2013a; Cerami et al., 2014; D'Ambrosio et al., 2014; Menke et al., 2014; Stoppel et al., 2014; Zhang et al., 2014; Raaphorst et al., 2015; Tavazzi et al., 2015). In total, 638 patients with ALS and 622 HCs were reported, and 210 coordinates were collected. Among the included studies, 21 studies described GM reductions in the patient group (Ellis et al., 2001; Chang et al., 2005; Grosskreutz et al., 2006; Agosta et al., 2007; Mezzapesa et al., 2007; Thivard et al., 2007; Grossman et al., 2008; Agosta et al., 2009; Minnerop et al., 2009; Canu et al., 2011; Senda et al., 2011; Cosottini et al., 2012; Tedeschi et al., 2012; Bede et al., 2013a; Cosottini et al., 2013; Meoded et al., 2013; Cerami et al., 2014; D'Ambrosio et al., 2014; Menke et al., 2014; Stoppel et al., 2014; Zhang et al., 2014; Devine et al., 2015; Tavazzi et al., 2015), and 6 studies found no significant changes between ALS patients and HCs (Abrahams et al., 2005; Sage et al., 2007; Minnerop et al., 2009; Roccatagliata et al., 2009; Luo et al., 2012; Raaphorst et al., 2015), only 1 study reported both increase and decrease of GM changes (Kwan et al., 2013). The demographic, clinical and technique details of included VBM studies were shown in Table 1.

**Table 1:** **Demographic, clinical and technique details of VBM studies included into the meta-analysis**.

**Study**	**Subjects (mean age, years)**		**Onset**		**Disease duration (months)**	**ALSFRS-R**	**Scanner(T)**	**Software**	**FWHM (mm)**	***P*-value**	**Coordinates**
	**Patients**	**HCs**	**Bulbar**	**Limb**							
Ellis et al., 2001	16 (55.1)	8 (55.8)	8	8	18.2	NA	1.5	AFNI	NA	*P* < 0.05 (uncorrected)	3
Chang et al., 2005	10 (49.9)	22 (44.5)	2	8	28.8	8^*^	1.5	SPM2	12	*P* < 0.05 (corrected)	9
Abrahams et al., 2005	11 (59.5)	12 (54.8)	5	6	20.3	NA	1.5	SPM2	NA	*P* < 0.01 (uncorrected)	0
Grosskreutz et al., 2006	17 (61)	17 (58)	6	11	24	40	1.5	SPM2	8	*P* < 0.05 (corrected)	16
Sage et al., 2007	28 (58.9)	26 (53.7)	7	21	14.6	39.7	3	SPM2	6	*P* < 0.05 (FWE corrected)	0
Agosta et al., 2007	25 (54.1)	18 (52.2)	9	19	39	29^*^	1.5	SPM2	8	*P* < 0.05 (corrected)	4
Mezzapesa et al., 2007	16 (58.6)	9 (51.8)	0	16	38.1	27.4^*^	1.5	SPM2	12	*P* < 0.001 (uncorrected)	13
Thivard et al., 2007	15 (51.8)	25 (44.9)	4	11	30.9	30	1.5	SPM2	8	*P* < 0.05 (FDR corrected)	19
Grossman et al., 2008	26 (NA)	16 (age-matched)	NA	NA	51.9	37	3	SPM2	12	*P* < 0.05 (FDR corrected)	11
Agosta et al., 2009	16 (55.2)	10 (47.9)	NA	NA	27	27	1.5	SPM2	12	*P* < 0.05 (corrected)	3
Minnerop et al., 2009	12 (59.5)	12 (59.6)	2	10	39.6	NA	1.5	SPM2	8	*P* < 0.05 (corrected)	0
Roccatagliata et al., 2009	14 (62.5)	12(60.6)	3	11	12.1	39.4	1.5	Freesurfer	NA	*P* < 0.05 (uncorrected)	0
Canu et al., 2011	23 (61)	14 (60)	3	20	24.4	33	1.5	SPM5	8	*P* < 0.001 (uncorrected)	6
Senda et al., 2011	17 (61.3)	17 (60.7)	5	12	38.4	36.7	3	SPM5	12	*P* < 0.001 (uncorrected)	5
Tedeschi et al., 2012	20 (60.7)	20 (62.1)	2	18	1-168	34.2	3	SPM8	8	*P* < 0.005 (uncorrected)	6
Luo et al., 2012	20 (45.3)	20 (47.1)	4	16	15.2	31.9	3	SPM8	8	*P* < 0.05 (FWE corrected)	0
Cosottini et al., 2012	20 (58)	16 (50.6)	3	17	20.1	38.2	1.5	FSL	Sigma = 3	*P* < 0.05 (FDR corrected)	21
Cosottini et al., 2013	18 (55.6)	18 (48.8)	4	14	20.3	38.8	1.5	FSL	Sigma = 3	*P* < 0.05 (FWE corrected)	4
Bede et al., 2013a	33 (60.8)	44 (60.2)	13	20	29.8	35.8	3	FSL	NA	*P* < 0.05 (FWE corrected)	5
Kwan et al., 2013	23 (55.8)	19 (58.7)	NA	NA	36	33.7	3	Freesurfer	NA	*P* < 0.001 (uncorrected)	7
Meoded et al., 2013	13 (51)	17 (59.2)	NA	NA	30	35.5	3	SPM8	10	*P* < 0.05 (FWE corrected)	1
Cerami et al., 2014	20 (59.1)	56 (61.9)	4	16	23.9	36.8	3	SPM8	8	*P* < 0.05 (FWE corrected)	11
D'Ambrosio et al., 2014	20 (61.5)	18 (60)	0	20	31.2	36.2	3	Freesurfer	10	*P* < 0.05 (Bonferroni corrected)	32
Menke et al., 2014	60 (61)	36 (53)	12	48	37	35	3	FSL	Sigma = 3	*P* < 0.05 (FWE corrected)	2
Stoppel et al., 2014	26 (60.4)	28 (60.1)	4	22	23.8	36.2	1.5	SPM8	4	*P* < 0.05 (FWE corrected)	18
Zhang et al., 2014	43 (53.5)	43 (51.8)	7	36	16.9	30.1^*^	3	FSL	Sigma = 3	*P* < 0.05 (FWE corrected)	5
Raaphorst et al., 2015	26 (60.7)	21 (60.7)	8	18	23.3	41.5	3	SPM5	8	*P* < 0.001 (FWE corrected)	0
Tavazzi et al., 2015	20 (54.5)	31 (47.9)	NA	NA	30.6	34.5^*^	1.5	FSL	Sigma = 3	*P* < 0.0001 (uncorrected)	7
Devine et al., 2015	30 (57.5)	17 (56)	4	26	26.3	39.5	3	FSL	Sigma = 4	*P* ≤ 0.05 (corrected)	2
Total	638	622									210

AFNI, analysis of functional neuroimages; ALS, amyotrophic lateral sclerosis; ALSFRS-R, revised ALS functional rating scale; FDR, false discovery rate; FSL, FMRIB software library; FWE, family-wise error; FWHM, full-width half maximum; HC, healthy control; NA, not available; SPM, statistical parametric mapping; T, Tesla; VBM, voxel-based morphometry.

* ALSFRS, ALS functional rating scale.

### Global GM volumes

Global GM volumes were obtained from only 4 studies including 89 patients with ALS and 72 HCs (Ellis et al., 2001; Mezzapesa et al., 2007; Zhang et al., 2014; Raaphorst et al., 2015). Heterogeneity analysis revealed no significant variance across studies (tau^2^ = 0.000, *Q* = 1.889, *P* = 0.596). The global GM volumes of patients with ALS were significantly decreased compared with those of HCs (655 ml vs. 686 ml, *Z* = −2.792, *P* = 0.005).

### Regional differences

**Figure 2:**
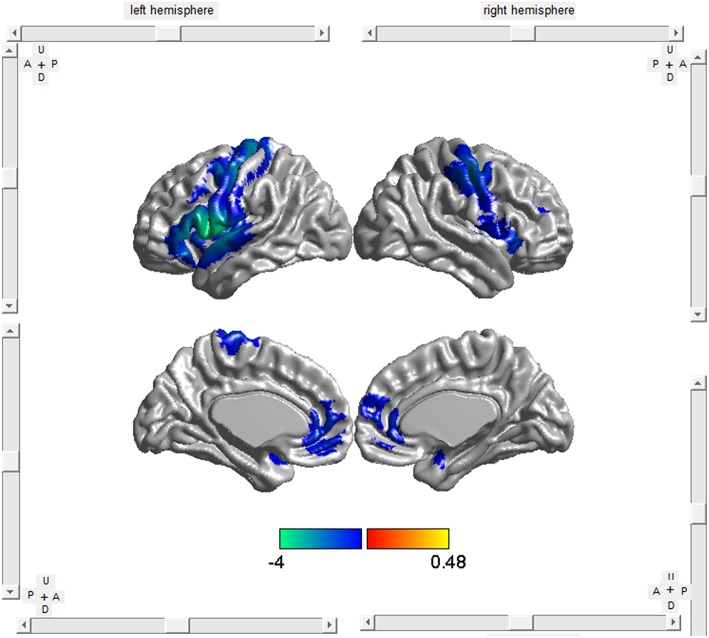
**Brat v1.0 (http://www.brainnetome.org/brat) software was used to visualize the anatomical distribution of GM atrophy in ALS compared with HCs**. Patients with ALS had significant GM reductions in bilateral Rolandic operculum, the right precentral gyrus, the left lenticular nucleus and the right anterior cingulate/paracingulate gyri. The color bar indicates the range of the SDM-Z values.

The included studies reported GM decrease at 209 coordinates and increase at 1 coordinate in ALS patients compared with HCs. As shown in Table 2, Figure 2, patients with ALS had significant GM reductions in the bilateral Rolandic operculum (larger on the left), the right precentral gyrus, the left lenticular nucleus (mainly the putamen) and the right anterior cingulate/paracingulate gyri from the SDM map with a threshold of *p* < 0.005 and cluster size ≥10 voxels. No GM increases were found.

**Table 2:** **Regional differences in GM volume between patients with ALS and HCs**.

**Region**	**Maximum**			**Clusters**		**Jackknife sensitivity analysis**
	**MNI coordinate (x,y,z)**	**SDM-Z**	***P*-values**	**No. of voxels**	**breakdown (No. of voxels)**	
Left rolandic operculum, BA 48	−50, 4, 2	−4.027	~0	7067	Left frontal gyrus (1301)	28 out of 29
					Left precentral gyrus (1182)	
					Left temporalgyrus (1083)	
					Left postcentral gyrus (894)	
					Left insula (702)	
					Corpus callosum (488)	
					Left rolandic operculum (459)	
					Left paracentral lobule (258)	
					Other/Undefined (624)	
Right precentral gyrus, BA 6	42, −16, 46	−3.062	0.000005186	1544	Right precentral gyrus (798)	29 out of 29
					Right postcentral gyrus (453)	
					Other/Undefined (293)	
Left anterior cingulate/paracingulate gyri	0,46,4	−2.395	0.000304461	873	Left anterior cingulate /paracingulate gyri (394)	28 out of 29
					Right anterior cingulate /paracingulate gyri (212)	
					Left superior frontal gyrus, medial (141)	
					Right superior frontal gyrus, medial (119)	
					Other/Undefined (7)	
Right lenticular nucleus, putamen, BA 48	28, 16, −4	−2.291	0.000449002	383	Right lenticular nucleus, putamen (173)	27 out of 29
					Right insula (64)	
					Right striatum (39)	
					Other/Undefined (107)	
Right rolandic operculum, BA 48	52, 6, 6	−1.843	0.003767371	43	Right rolandic operculum (35) Other (8)	20 out of 29

ALS, amyotrophic lateral sclerosis; BA, Brodmann; GM, gray matter; HC, healthy control; MNI, Montreal Neurological Institute; SDM, signed differential mapping.

### Sensitivity analyses

A whole-brain Jackknife sensitivity analysis was conducted to test the replicability of the results, which consists of repeating the Mean analysis 29 times but systematically removing one different study each time to recalculate the stability of the remaining studies. As shown in Table 3, the analysis indicated GM reductions in the left Rolandic operculum, the right precentral gyrus, the left lenticular nucleus and the right anterior cingulate/paracingulate gyri were highly replicable because they were preserved in all or almost the combinations. GM decreases in the right Rolandic operculum failed to emerge in nine of the combinations, which we conservatively did not consider as a significant finding.

**Table 3:** **Subgroup analyses of included studies**.

**Subgroups**	**Left rolandic operculum, BA 48**	**Right precentral gyrus, BA 6**	**New findings**
Studies using SPM (*n* = 18)	No	Yes	Left inferior frontal gyrus
Studies using FWHM of 6–8 mm (*n* = 15)	No	Yes	Left superior temporal gyrus
Studies using GM volume (*n* = 23)	Yes	Yes	Left superior temporal gyrus
Studies correcting for multiple comparisons(*n* = 20)	Yes	Yes	Left inferior/superior frontal gyrus

BA, Brodmann; FWHM, full-width half maximum; GM, gray matter; SPM, statistical parametric mapping.

### Analyses of subgroups

When the analyses were repeated and limited to methodologically homogenous groups of studies, GM atrophy in the left Rolandic operculum and right precentral gyrus remained largely or totally unchanged. While GM decreases in other regions identified in the main results did not survive in the subgroups analyses, additional significant cluster in the left inferior/superior frontal gyrus and left superior temporal gyrus emerged (Table 3).

### Meta-regression

**Figure 3:**
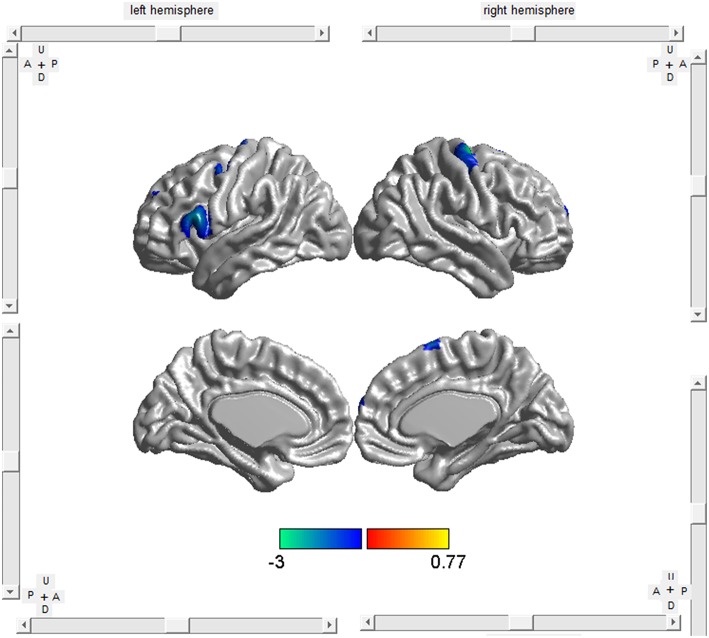
**Meta-regression analysis revealed that higher symptom severity was associated with more GM atrophy in the right precentral gyrus (BA 6) and the left inferior frontal gyrus (BA 44) in ALS patients**.

**Figure 4:**
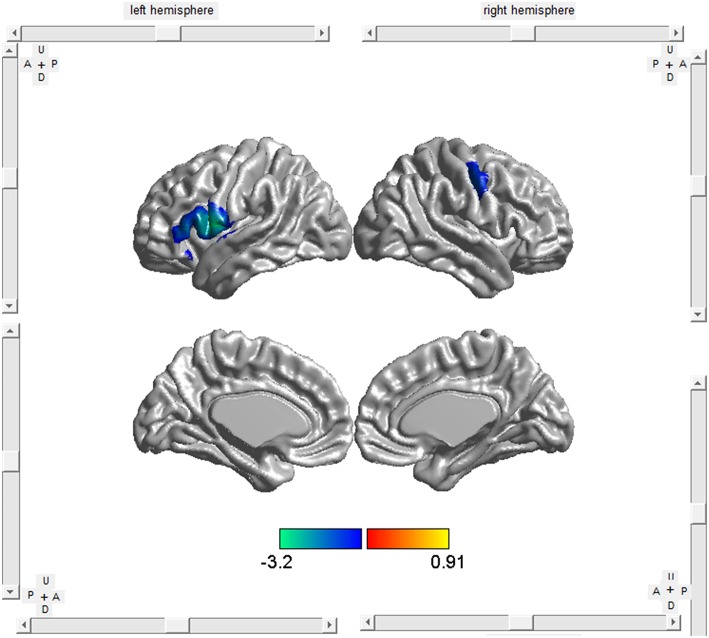
**Meta-regression analysis revealed that longer disease duration was associated with more GM atrophy in the left frontal aslant tract and the right precentral gyrus (BA 4) in ALS patients**.

The meta-regression analyses showed that higher symptom severity (proportional scores, ALSFRS scores available in 5 studies and ALSFR-R scores available in 21 studies) was associated with decreased GM in the right precentral gyrus and the left inferior frontal gyrus (Table 4, Figure 3). A longer disease duration correlated with more GM atrophy in the left frontal aslant tract and the right precentral gyrus in patients with ALS (Table 4, Figure 4).

**Table 4:** **Meta-regression analyses of disease severity and duration on GM abnormalities in patients with ALS**.

**Regions**	**Peak MNI coordinate (x,y,z)**	**SDM-Z**	**P-values**	**No. of Voxels**
**DISEASE SEVERITY**				
Right precentral gyrus, BA6	32, −14, 62	−3.025	0.000030994	339
Left inferior frontal gyrus, BA 44	−54, −16, 16	−2.410	0.001145720	218
**DISEASE DURATION**				
Left frontal aslant tract	−54,4,10	−3.216	~0	690
Right precentral gyrus, BA4	46, −12,40	−2.373	0.000165164	327

ALS, amyotrophic lateral sclerosis; BA, Brodmann; GM, gray matter; MNI, Montreal Neurological Institute; SDM, signed differential mapping.

## Discussion

The present study updated the meta-analysis of GM changes in patients with ALS and yielded some new findings. Compared with the previous SDM meta-analysis (Chen and Ma, 2010), the main strengths of our study included: (1) the application of the latest version of the SDM software which is more efficient and precise than its previous ones; (2) a larger sample size since a sufficient number of high-quality studies have only recently become available; (3) the unbiased inclusion of published studies even if their results were negative; (4) more clear definition of the participants as we excluded the comparisons containing a subgroup of ALS-FTD; (5) further subgroup and meta-regression analyses made the current meta-analysis more comprehensive.

By analyzing data sets from 29 VBM studies, the meta-analysis demonstrated consistent GM atrophy in the left Rolandic operculum, the right precentral gyrus, the left lenticular nucleus and the right anterior cingulate/paracingulate gyri in individuals with ALS compared to HCs. These findings were highly replicable as verified by the jackknife sensitivity analysis. However, when methodologically homogeneous studies were analyzed separately, only part of the results survived and revealed new findings of GM reductions in the left inferior/superior frontal gyrus and left superior temporal gyrus. Further meta-regression analyses indicated higher symptom severity by proportional functional scores was associated with decreased GM in the right precentral gyrus and the left inferior frontal gyrus. Moreover, increased disease duration was associated with more GM atrophy in the left frontal aslant tract and the right precentral gyrus.

In accord with the previous meta-analysis (Chen and Ma, 2010), we detected regional GM loss over the whole brain in the right precentral gyrus, and the result remained totally unchanged in the Jackknife sensitivity analyses and the subgroup analyses, in contrast to the reproducibility being only 3 out of 5 in the earlier study. The motor cortex has long been a main focus of ALS studies and advanced neuroimaging studies have demonstrated substantial changes in this area in patients with ALS (Foerster et al., 2013): structural imaging and proton magnetic resonance spectroscopy (^1^H-MRS) studies have provided evidence of neuronal degeneration, through measures showing reduced cortical thickness and reductions in N-acetylaspartate/creatine (NAA/Cr) ratio or NAA levels (Rule et al., 2004; Han and Ma, 2010; Agosta et al., 2012; Verstraete et al., 2012); functional magnetic resonance imaging (fMRI) studies (Turner and Kiernan, 2012) have indicated that increase in motor network connectivity could be a manifestation of loss of neuronal inhibitory function, which is consistent with the decreased GABA concentration in the motor cortex of ALS patients found in PET studies (Foerster et al., 2012). Furthermore, meta-regression analyses showed that studies that included patients with more severe ALS and longer duration were significantly more likely to report decrease GM in these regions.

It is noteworthy that the atrophy pattern presented asymmetry in ALS with only the right precentral gyrus being selectively impaired. In line with our findings, Cosottini et al. (2012) found a prevalent atrophy in the nondominant right hemisphere in their VBM study, and an interhemispheric asymmetry was also revealed by their fMRI data that showed more active hyperactivation of the fronto-parietal circuit during the motor task executed with right hand than that executed with the left hand. The researchers assumed that the residual motor function of ALS patients correlated with an enhanced activation of the fronto-parietal circuit mostly on the left hemisphere in order to preserve the more sophisticated motor function of the dominant hand (right hand in their study), therefore the asymmetric distribution of this circuit and cortical atrophy suggested a higher vulnerability of the non-dominant hemisphere to neurodegenerative process. Zhang et al. (2014) proposed another hypothesis that side of limb-onset can predict laterality of GM loss in ALS patients, which was demonstrated in the subgroup comparisons that the motor cortex in the contralateral hemisphere of the initially involved limb was most affected, thus the left predominant GM loss in the whole group was mainly reflective of the abnormalities caused by the subpopulation of patients with right limb-onset. However, Devine et al. (2015) described that subjects with ALS showed disproportionate atrophy of the dominant (left) motor cortex hand area, irrespective of the side of first limb weakness, and that increased complexity of left hemispheric motor networks in right-handers resulting in greater susceptibility to neurodegeneration might be one of the explanations. Although, the increased statistical power of the current meta-analysis make our results more reliable across different studies, still our data did not allow to fully understand the pathophysiology of this phenomenon since the heterogeneous disease progression rate in individual ALS patients will increase or decrease the asymmetry dominated by motor symptoms and add to the complexity of this disease, which require further exploration.

For the first time, we found that patients with ALS had bilateral regional GM atrophy in the Rolandic operculum (larger on the left), and the result remained largely unchanged in the Jackknife sensitivity analyses. The Rolandic operculum is located on the surface of the posterior subcentral gyrus within the Sylvian fissure and is a part of the articulated language output network (Kertesz, 1995; Eickhoff, 2005; Eickhoff et al., 2006). Individuals with lesions of this area might get articulatory disorders or phonemic fluency deficiencies (Tonkonogy and Goodglass, 1981; Behroozmand et al., 2015; Biesbroek et al., 2015). So we speculated that atrophy in the Rolandic operculum might be another important reason to explain the dysarthria symptom in ALS patients besides the damage in brainstem motor nuclei. Consistent with our findings, a MRS study revealed that the NAA/Cr ratio was significantly lower in the Rolandic operculum in the patient group, suggesting neuronal degeneration in this region (Verma et al., 2013). Alterations in the Rolandic operculum have been noted in Parkinson's disease as well (New et al., 2015), with the severity of speech-motor impairments correlating with the left Rolandic operculum connectivity in the voice network, which lends further support to our theory.

Of note, GM atrophy in the left Rolandic operculum only survived part of the subgroup analyses, and an additional significant cluster in the left inferior/superior frontal gyrus and left superior temporal gyrus emerged. Neuroimaging studies report the coordinates of the “voxel” where the difference between patients and controls is maximum from each cluster of significant differences, which means the breakdown of a cluster all have contributed to the abnormalities (Table 3; Radua and Mataix-Cols, 2009). Thus, though subgroup analyses with reduced sample size may reveal altered peak coordinates, it was still a reflection of the same functional area adjacent to each other, consisting of left Rolandic operculum, inferior frontal gyrus and superior temporal gyrus in this case (Figure 2). These findings also correspond to the regional pattern of the extra-motor changes reported previously. In small samples of ALS patients without dementia, cerebral activation in the frontal and temporal regions is altered when undertaking executive and language tasks (Abrahams et al., 2004; Goldstein et al., 2011). The left inferior frontal gyrus is critical for verbal fluency, which is a sensitive marker of cognitive dysfunction in ALS patients (Goldstein and Abrahams, 2013). Besides, the meta-regression also showed that more GM atrophy in the left inferior frontal gyrus correlated with greater severity of the disease.

Another notable finding was GM reductions detected in anterior cingulate cortex (ACC) and the deep gray nuclei, though they failed to emerge in the subgroup analyses. This might be due to a smaller sample size, since there were not so many voxels in these two regions in the main analyses. The ACC is an important component of the limbic system, and could act as a monitoring system in the executive function. This area has also been implicated in our study and in a wide range of functional neuroimaging studies in ALS. In fMRI studies employing extra-motor paradigms, hyperactivity in the ACC was found during stroop tasks and socioemotional stimuli tasks (Goldstein et al., 2011; Passamonti et al., 2013), and hypoactivity in the ACC was found during letter fluency task (Raaphorst et al., 2014). Reduced response in the ACC is also found to be associated with hands movement imagery, which might reflect disruption of the normal networks associated with motor imagery (Stanton et al., 2007). Interestingly, ALS patients with greater UMN involvement had more robust activation of ACC and right caudate nucleus than those with greater LMN involvement when performing hand movements (Tessitore et al., 2006). The enhanced activation might be interpreted as a compensation for the structural damage in ALS patients with greater cortical dysfunction, but this increase will be exhausted with disease progression. Another interesting study revealed that atrophy in anterior cingulate and paracingulate gyrus was common across ALS, ALS-FTD and behavioral variant FTD, suggesting that ALS and FTD lie on a pathological continuum (Lillo et al., 2012).

The components of the deep gray nuclei have important connections to the motor network and prefrontal cortices. Despite the ample clinical and postmortem evidence of basal ganglia pathology in ALS (Brettschneider et al., 2013), most neuroimaging studies focus on cortical gray and subcortical white matter pathology, whereas only a few volumetric changes involving the deep gray nuclei have been described in the ALS literature. Agosta et al. (2009) described atrophy in the right basal ganglia in ALS; moreover, rapidly progressing patients showed more substantial GM loss in the right putamen compared with both HCs and non-rapidly progressing cases. Machts et al. (2015) demonstrated a gradient of incremental basal ganglia pathology across the ALS-ALS/FTD spectrum. They also further reported that the putamen atrophy in ALS was predominantly confined to the dorsal aspect of the structure. But it should also be noted that other studies of subcortical pathology in ALS did not demonstrate changes in the putamen (Bede et al., 2013b; Westeneng et al., 2015), probably due to their unsegregated patient cohorts. Our results provide further neuroimaging evidence for basal ganglia involvement in ALS.

There are several limitations of the methodology. Firstly, though the SDM method is able to provide an excellent control of false positives, it is difficult to completely avoid false-negative results. Next, this approach is based on pooling of summarized coordinates rather than raw statistical brain maps, which may result in less accuracy. Furthermore, methodological differences of included VBM studies, such as preprocessing protocols, smoothing kernels, statistical thresholds, and adoption of GM density or volume or cortical thickness, cannot be entirely ruled out, though we conducted the subgroup analyses. Finally, the meta-regression results should be taken with some caution because of the variability in the data. The included studies also have their limitations. On one hand, though we excluded the comparisons containing a subgroup of ALS-FTD, a high prevalence of executive dysfunction was present in patients with ALS without dementia screened by the frontal assessment battery (Barulli et al., 2015), and most of the original researches did not offer a neuropsychological assessment of the patients; therefore we were unable to conduct subgroup analyses or meta-regression analyses based on the neuropsychological or cognitive symptoms, especially the presence of executive dysfunction, because of the limited data. On another hand, patients with bulbar-onset or limb-onset were not studied separately, which made it impossible to evaluate association between GM atrophy and site of onset of the disease, especially the implication of Rolandic operculum involvement in bulbar-onset patients.

In summary, using the ES-SDM method of meta-analysis, we identified consistent GM reductions in patients with ALS, mainly located in the right precentral gyrus, the left Rolandic operculum, the right lenticular nucleus and the right anterior cingulate/paracingulate gyri; the right precentral gyrus and the left inferior frontal gyrus might be potential anatomical biomarkers to evaluate the severity of the disease. These changes suggest that ALS is a complex degenerative disease involving language output network, limbic system, and deep gray nuclei, besides the motor system. The mechanism of asymmetric atrophy of the motor cortex and the implication of Rolandic operculum involvement in ALS need to be further elucidated in future clinical and neuroimaging studies. Longitudinal VBM studies with quantitative neuropsychological tests and investigations exploring the GM atrophy and site of onset of the disease are also worthwhile.

## Author contributions

DS: Literature search, Statistical Analysis, Writing of the first draft. LC: Research Organization, Manuscript review and Critique. JF: Literature search, Data extraction. BC: Data extraction, Statistical Analysis. DL: Statistical analysis review and critique. HT: Statistical analysis review and critique.

## Funding

This work was supported by Sino-Germany Science Research Foundation (GZ876, http://www.sinogermanscience.org.cn). The founder is Chinesisch-Deutsche Zentrum für Wissenschaftsförderung.

### Conflict of interest statement

The authors declare that the research was conducted in the absence of any commercial or financial relationships that could be construed as a potential conflict of interest.
